# Genomic characterization of *Lactobacillus fermentum* DSM 20052

**DOI:** 10.1186/s12864-020-6740-8

**Published:** 2020-04-29

**Authors:** Katelyn Brandt, Matthew A. Nethery, Sarah O’Flaherty, Rodolphe Barrangou

**Affiliations:** 1 Functional Genomics Graduate Program, North Carolina State University, Raleigh, NC 27695 USA; 2 Department of Food, Bioprocessing and Nutrition Sciences, North Carolina State University, Raleigh, NC 27695 USA

**Keywords:** *Lactobacillus*, *Fermentum*, Comparative genomics, CRISPR

## Abstract

**Background:**

*Lactobacillus fermentum,* a member of the lactic acid bacteria complex, has recently garnered increased attention due to documented antagonistic properties and interest in assessing the probiotic potential of select strains that may provide human health benefits. Here, we genomically characterize *L. fermentum* using the type strain DSM 20052 as a canonical representative of this species.

**Results:**

We determined the polished whole genome sequence of this type strain and compared it to 37 available genome sequences within this species. Results reveal genetic diversity across nine clades, with variable content encompassing mobile genetic elements, CRISPR-Cas immune systems and genomic islands, as well as numerous genome rearrangements. Interestingly, we determined a high frequency of occurrence of diverse Type I, II, and III CRISPR-Cas systems in 72% of the genomes, with a high level of strain hypervariability.

**Conclusions:**

These findings provide a basis for the genetic characterization of *L. fermentum* strains of scientific and commercial interest. Furthermore, our study enables genomic-informed selection of strains with specific traits for commercial product formulation, and establishes a framework for the functional characterization of features of interest.

## Background

*Lactobacillus* are low-GC, microaerophilic, Gram-positive microorganisms that are members of the lactic acid bacteria (LAB) group [1]. They are considered ubiquitous in nature and many species and strains have received Generally Recognized as Safe (GRAS) or Qualified Presumption of Safety (QPS) status [2]. They have had a large impact on the food manufacturing, human health, and biotechnology industries. Their ability to spontaneously ferment foods and produce lactic acid has ingratiated lactobacilli into the food manufacturing process, specifically as starter cultures to produce yogurt, cheese, and fermented vegetables [3]. Several strains of *Lactobacillus* are used as probiotics, defined as “live microorganisms which when administered in adequate amounts confer a health benefit on the host” [4, 5]. Several species are widely studied and utilized, such as *Lactobacillus acidophilus, Lactobacillus gasseri,* and *Lactobacillus rhamnosus,* with specific strains heavily studied and boasting probiotic functionalities such as NCFM and LGG*.* Additionally, *Lactobacillus* serves as a valuable source of clustered regularly interspaced short palindromic repeats (CRISPR) and associated proteins (Cas), which may be repurposed for a diversity of applications, including the development of genome editing tools [6]. Recently, there has been an increased interest in assessing the potential of various *Lactobacillus* species and strains for the development of new functional foods, biotechnology tools, and next-generation probiotics. *Lactobacillus fermentum* is one such candidate species being examined for its potential use.

A survey of metagenomic study data using Integrated Microbial Next Generation Sequencing (IMNGS) [7] revealed that the most common metagenomes for *L. fermentum* are fermentation and human gut metagenomes. This implies use or effectiveness in food manufacturing and human health. Various studies over the years have looked at the ability of *L. fermentum* to serve as a potential probiotic or biotechnology tool beyond its current uses in food manufacturing. *L. fermentum* is known for its biofilm formation phenotype and has been studied as a potential biosurfactant in numerous capacities, including for the sterilization of surgical implants [8, 9]. Some strains of *L. fermentum* have been shown to inhibit pathogens through the production of bacteriocins and antifungal metabolites [10, 11]. This, combined with the ability to survive bile salts and lower cholesterol, suggests that some *L. fermentum* strains may have some potential for probiotic applications [12, 13]. In fact, two *L. fermentum* strains, ME-3 and CECT 5716, have been characterized for probiotic attributes. *L. fermentum* ME-3 has antioxidant properties as well as demonstrated antimicrobial capabilities against Gram-negative organisms, *Enterococcus,* and *Staphylococcus aureus* [14]. *L. fermentum* CECT 5716 has the ability to modulate immune responses of host organisms [15].

Despite the interest in *L. fermentum*, there have been relatively few studies overall for this species, especially regarding the type strain DSM 20052 (ATCC 14931). The type strains serve as the reference for the species, and as such established a foundation and reference for species-wide comparisons. Lack of study regarding *L. fermentum* DSM 20052 has led to relatively limited knowledge with regards to genomic diversity at the species level. One study compared five *L. fermentum* strains but did not include the type strain [16]. In order to fully leverage the potential of *L. fermentum,* we should first assess genetic species diversity and identify strains of reference and interest. In this study, we evaluated the type strain DSM 20052 through comparative genomic analyses against 37 strains to establish the diversity of the overall species.

## Results

### Complete genome sequence of *L. fermentum* DSM 20052

A draft genome for *L. fermentum* DSM 20052 was previously deposited at NCBI in 2009 and updated in 2017 as NZ_ACGI, which contained 74 contigs. We re-sequenced and completed the genome sequence and generated a single contig (1.89 Mb). The genomic traits for *L. fermentum* DSM 20052 can be found in Table 1. The genome size is 1.89 Mb with a GC content of 52.5%. We identified no plasmids in *L. fermentum* DSM 20052. Next, we annotated the genome using RAST, which identified 1900 coding sequences and 73 RNAs (15 rRNA and 58 tRNA). Using EggNOG, we assigned COG groups to the ORFs (open reading frames) encoded throughout the genome sequence. Of the 1900 coding sequences, 1237 were given a COG designation. The largest COG group was the [S] group (15% of assigned coding sequences), or the unknown function group [17] Of note, closer examination of the genome revealed several loci of interest, including a putative *exopolysaccharide* locus and one CRISPR-Cas (CRISPR associated) locus. Additionally, there were several annotated transposases and mobile genetic elements (MGE). As the spread of antibiotic resistance is of growing concern, we next analyzed *L. fermentum* DSM 20052 for any antibiotic resistance genes using ResFinder. We found none, which is consistent with the aforementioned GRAS status of this species.

**Table 1:** Genomes List

Strain	Sequence Length	GC%	#Sequences	#Plasmids	Accession	Isolation
DSM 20052	1,887,974	52.50%	1	0	CP040910	Fermented beets
MTCC 25067	1,954,694	51.50%	1	1	NZ_AP017973.1	Fermented Milk
VRI-003	1,949,297	52.10%	1	0	CP020353.1	Commercial Probiotic
IMD0 130,101	2,089,202	51.50%	1	0	LT906621.1	Sourdough
IFO 3956	2,098,685	51.50%	1	0	NC_010610	Fermented plant material
CECT 5716	2,100,449	51.50%	1	0	NC_017465	Human milk
F-6	2,064,620	51.70%	1	0	NC_021235	Unknown
3872	2,297,851	50.70%	1	1	NZ_CP011536	Milk
NCC2970	1,949,874	52.20%	1	0	NZ_CP017151	Unknown
47–7	2,098,685	52.50%	1	0	NZ_CP017712	Unknown
SNUV175	2,176,678	51.50%	1	3	NZ_CP019030	Human vagina
FTDC 8312	2,239,921	51.00%	1	0	NZ_CP021104.1	Human feces
LAC FRN-92	2,063,606	51.80%	1	0	NZ_CP021790.1	Human oral
LfQi6	2,098,510	52.50%	1	0	NZ_CP025592.1	Human microbiome
HFB3		51.80%	7	0	LJFJ00000000.1	Human gut
28–3-CHN		52.20%	42	0	NZ_ACQG00000000	Human
39		51.60%	55	0	NZ_LBDG00000000	Unknown
L930BB		52.10%	72	0	NZ_CBUR000000000	Human intestine
222		52.10%	73	0	NZ_CBZV000000000	Cocoa bean
RI-508		52.20%	74	0	NZ_MKGE00000000.1	Cacao bean fermentation
MD IIE-4657		52.30%	74	0	NZ_PTLW00000000.1	Silage
S6		52.30%	82	0	NZ_FUHZ00000000.1	Unknown
S13		52.30%	85	0	NZ_FUHY00000000.1	Unknown
90 TC-4		51.90%	93	0	NZ_LBDH00000000	Unknown
SHI-2		52.10%	93	0	NZ_NJPQ00000000.1	Human saliva
DSM 20055		52.40%	102	0	NZ_JQAU00000000	Human Saliva
UC0-979C		51.90%	108	0	NZ_LJWZ00000000	Human gastric
279		52.00%	108	0	NZ_PGGI00000000.1	Human feces
103		51.80%	110	0	NZ_PGGE00000000.1	Human cecum
311		51.80%	111	0	NZ_PGGJ00000000.1	Human feces
MTCC 8711		49.70%	116	7	NZ_AVAB00000000	Yogurt
CECT 9269		51.70%	129	0	NZ_OKQY00000000.1	Tocosh
LfU21		51.70%	131	0	NZ_PNBB00000000.1	Human feces
NB-22		51.80%	137	0	NZ_AYHA00000000	Human vagina
NCDC 400		51.60%	138	0	NZ_PDKX00000000.1	Curd
BFE 6620		52.10%	149	0	NZ_NIWV00000000.1	Gari
779_LFER		52.10%	169	0	NZ_JUTH00000000	Unknown
Lf1		52.60%	250	0	NZ_AWXS00000000	Human gut

Genomic features of 38 *L. fermentum* strains used in this study

### *L. fermentum* species genetic diversity

**Fig. 1:**
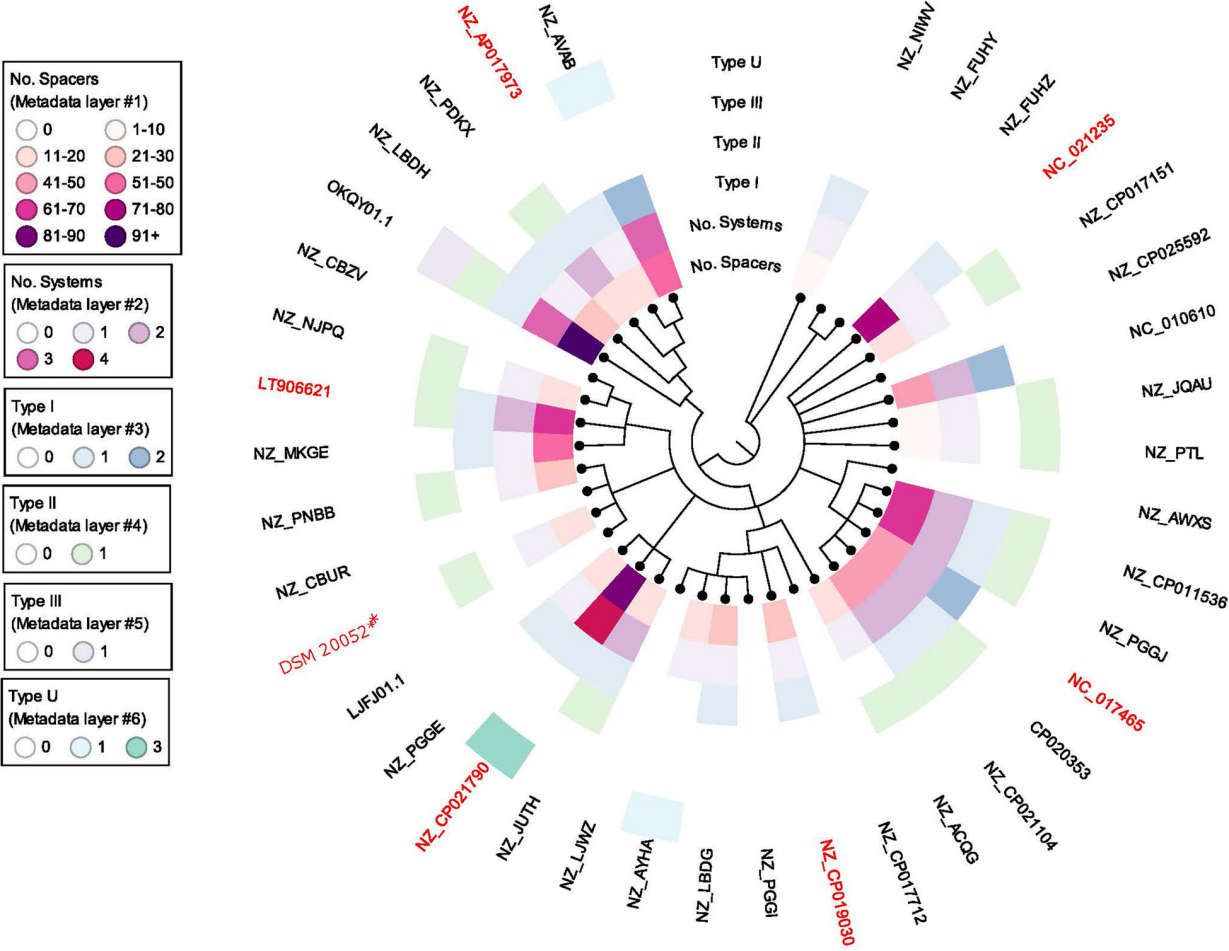
*Lactobacillus fermentum* Phylogeny. Phylogenetic tree generated of 38 *L. fermentum* strains using RAxML based on the nucleotide alignment of *phosphoglucomutase.* DSM 20052 is indicated by a red asterisk (*). Genomes in red are used for subsequent BRIG comparisons. Rings refer to CRISPR-Cas analyses performed on the genomes and are (from inside out): number of spacers in the genome, number of total systems in the genome, number of Type I systems in the genome, number of Type II systems in the genome, number of Type III systems in the genome, and number of undefined Types in the genome. Ring legends are in the insets. Strain names can be found in Table 1

With a complete genome sequence for the type strain, we next determined how DSM 20052 compares to other *L. fermentum* strains and carried out comparative genomic analyses. Thirty-seven strains, in addition to DSM 20052 (Table 1), were chosen for comparative analysis using the glycolysis gene *phosphoglucomutase* (Fig. 1). Nine clades were identified in the phylogeny. *L. fermentum* DSM 20052, highlighted by a red asterisk (*), was found to be a part of a four-member clade that included the strains HFB3 (LJFJ01.1), L930BB (NZ_CBUR), and Lfu21 (NZ_PNBB). Interestingly, HFB3 and Lfu21 were isolated from human fecal samples, while L930BB was isolated from a human colon biopsy (Table 1).

**Fig. 2:**
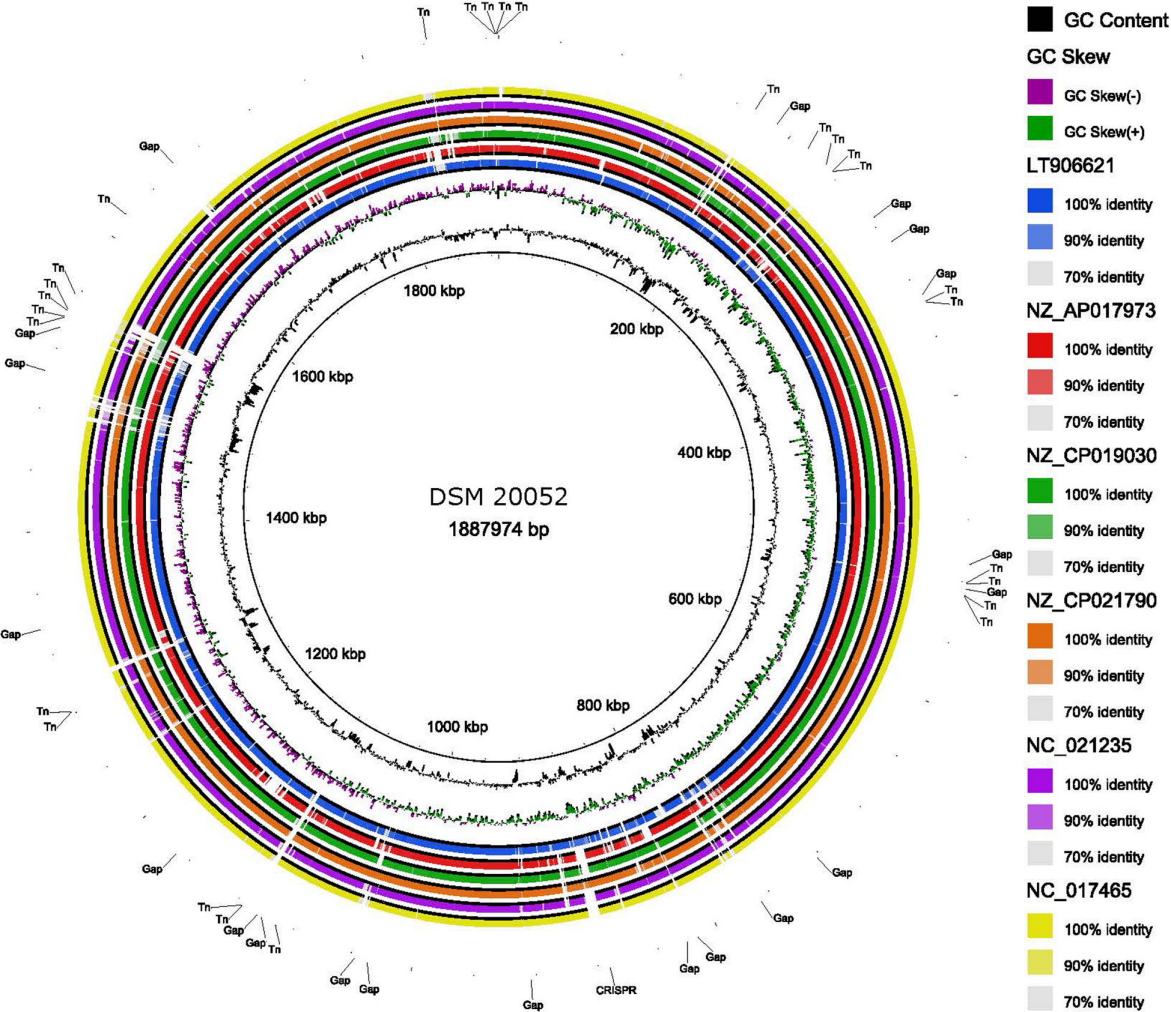
BRIG Analysis. BRIG alignment of seven *L. fermentum* genomes with DSM 20052 as the reference. The innermost ring denotes genome location. The other rings and color specifications can be found to the right of the ring image. Transposases, CRISPR genes, and minor assembly gaps are annotated outside of the rings. Strain names can be found in Table 1

**Fig. 3:**
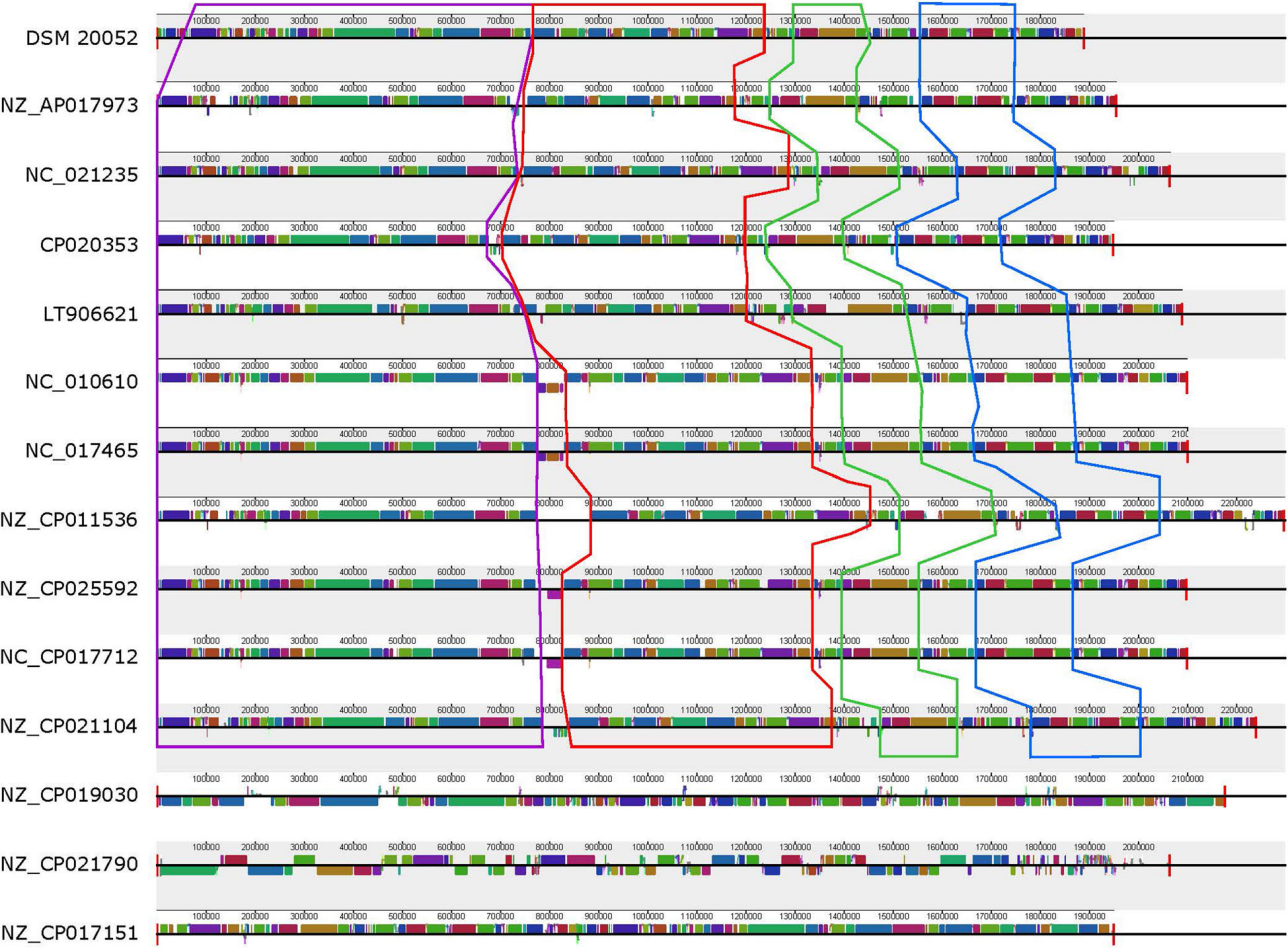
Whole Genome Comparisons. MAUVE alignment of all complete *L. fermentum* genomes with DSM 20052 set at the reference. Grouped blocks of similarity are boxed. Strain names can be found in Table 1

Next, we selected six strains to perform whole genome comparisons with *L. fermentum* DSM 20052. The genomes chosen for further analyses were: LT906621 (IMDO 130101, sourdough), NZ_AP017973 (MTCC 25067, fermented milk), NZ_CP019030 (SNUV175, human vagina), NZ_CP021790 (LAC FRN-92, human oral), NC_021235 (F-6, unknown), and NC_017465 (CECT 5716, human milk). These genomes were chosen as a representative set of the phylogeny generated in Fig. 1 and are highlighted in red. They all contain a single contig or closed genome and range in size from 1.95 Mb to 2.18 Mb. GC content for each strain was ~ 51% (Table 1). MTCC 25067 and SNUV175 both carry plasmids. Using these six genomes in addition to DSM 20052, whole genome analysis was carried out with BRIG (Fig. 2). From the BRIG analysis, there are several islands in *L. fermentum* DSM 20052 that do not occur within the other genomes. These islands at approximately 180 kbp, 760 kbp, and 1550 kbp also correlate with GC dips. Further examination of these three islands did not reveal loci of note (Additional files 1, 2, 3), but several transposases in or around each island were identified (Fig. 2). There are several smaller GC dips throughout the genome that correlate to either transposases or minor assembly gaps. There were no GC spikes observed. Another island of note is the CRISPR locus of *L. fermentum* DSM 20052, which only had a homolog in LT906621, annotated at 880 kbp. Finally, the GC skew switches around 50 kbp and 1090 kb. Due to the large presence of transposases, we next used MAUVE to determine gene synteny amongst *L. fermentum* genomes (Fig. 3). For this analysis, we used all genomes consisting of a single contig/closed genome, in addition to the strains used for the BRIG analysis (Table 1). Examination of the MAUVE alignment showed several small blocks of synteny among the strains, in contrast to the expected large blocks of similarity. These small blocks generated by MAUVE could be combined into larger regions of synteny (outlined in boxes). In addition, there were several rearrangements observed, especially for genomes NZ_CP019030 (SNUV175, human vagina), NZ_CP021790 (LAC FRN-92, human oral), and NZ_CP017151 (NCC2970, unknown) (Fig. 3). These smaller blocks of synteny and genome rearrangements could be due to the presence of transposons in the genomes.

### CRISPR-Cas immune systems diversity

Next, we examined the occurrence and diversity of CRISPR-Cas systems in *L. fermentum* across 38 strains (Fig. 1). Potential CRISPR loci were identified using the CRISPR recognition tool (CRT) and then hand-curated. Types I, II, and III were all identified in *L. fermentum*. Several loci did not contain the complete *cas* complement due to draft genome sequences or transposons and were thus labelled unknown (Fig. 1). Of the 38 strains analyzed, 71.8% encoded putative CRISPR-Cas systems. 53.8% of the strains analyzed contained a Type I system, 41.0% a Type II system, and 2.56% a Type III system. This is relatively hypervariable within a species, given the very high relative level of occurrence, and the absence of a single CRISPR-Cas system type that is widely shared across the species is noteworthy. Interestingly, one strain (OKQY01.1), contained a Type I, II, and III system, which is very rare in bacteria. This was the only strain with over 91 spacers in its genome (Fig. 1).

**Fig. 4:**
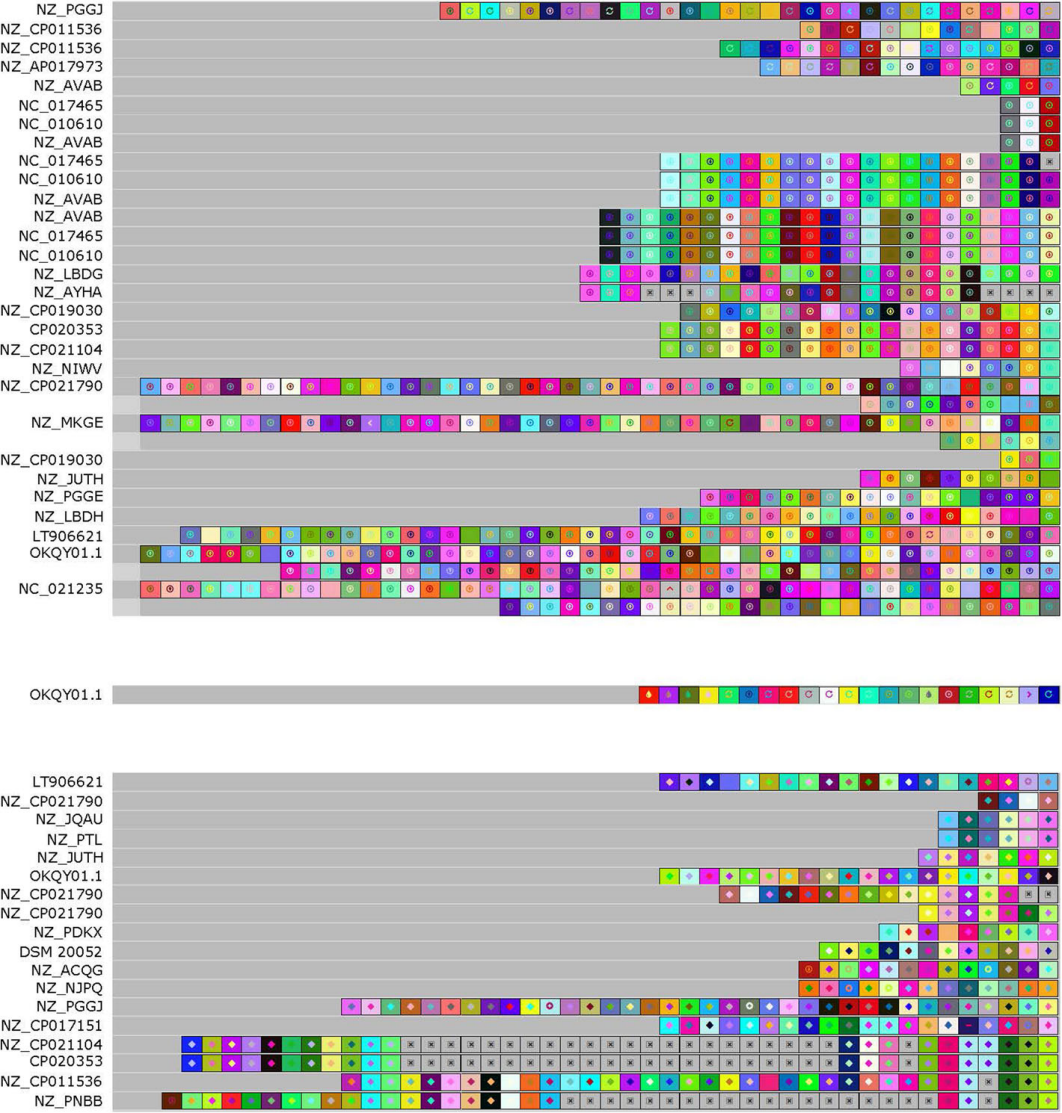
CRISPR Spacer Visualization. Visualization of CRISPR spacers for 38 *L. fermentum* strains using CRISPRviz. Spacers for putative Type I loci are on the top, with Type III loci in the middle, and Type II loci on the bottom. Ancestral spacers are on the right-hand side of the figure. Strain names can be found in Table 1

**Fig. 5:**
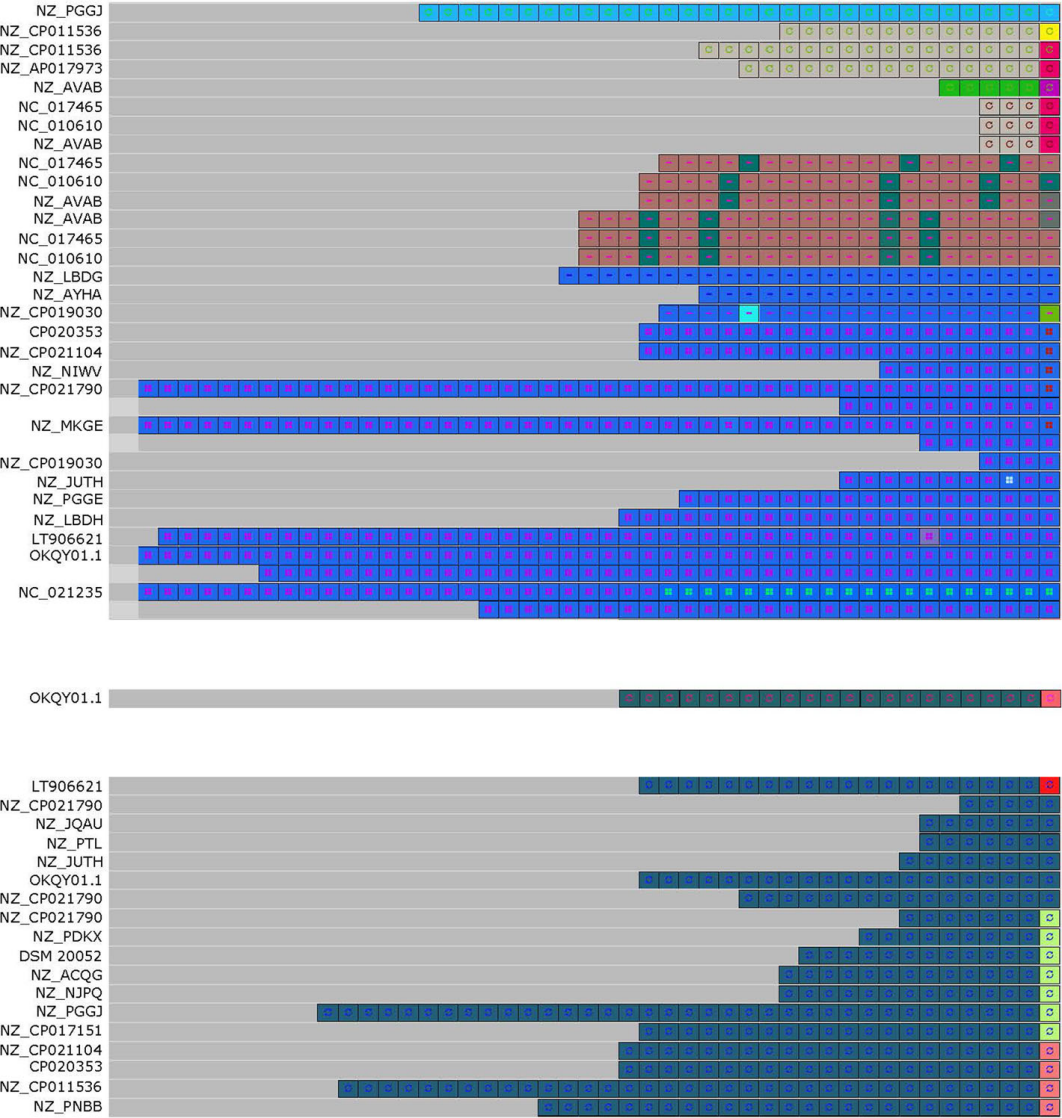
CRISPR Repeat Visualization. Visualization of CRISPR repeats for 38 *L. fermentum* strains using CRISPRviz. Repeats for putative Type I loci are on the top, with Type III loci in the middle, and Type II loci on the bottom. Ancestral repeats are on the right-hand side of the figure. Strain names can be found in Table 1

We then used CRISPRviz to compare the spacer content and, presumably, the history of the strains (Fig. 4). Type I, II, and III spacers grouped based on CRISPR-Cas systems. As expected, Type I systems encoded for a greater number of spacers than that of the Type II systems [18]. The spacers in *L. fermentum* as a whole were very diverse and we were unable to identify common ancestral spacers for the majority of the strains. Three genomes (NZ_AVAB, NC_010610, and NC_017465) had the most similar spacer arrays, only differing by one or two spacers in any of their Type I loci (Fig. 4). Interestingly, each of these three genomes belonged to a different clade in the *L. fermentum* phylogeny (Fig. 1). Of those with Type II systems, the genomes NZ_CP021104, CP020353, NZ_CP011536, and NZ_PNBB shared some spacers, but also each had a great deal of unique spacers (Fig. 4). Specifically, they shared a common ancestry and some newer additions; the main deviation was the large number of additional spacers in NZ_CP011536 (Fig. 4). Interestingly, these genomes were a part of the same clade, with the exception of NZ_PNBB (Fig. 1). A few other genomes, such as NZ_JQAU and NZ_PTL, also shared common spacers amongst each other. Even though the spacers varied widely, the repeats in *L. fermentum* did group with high similarity (Fig. 5).

**Fig. 6:**
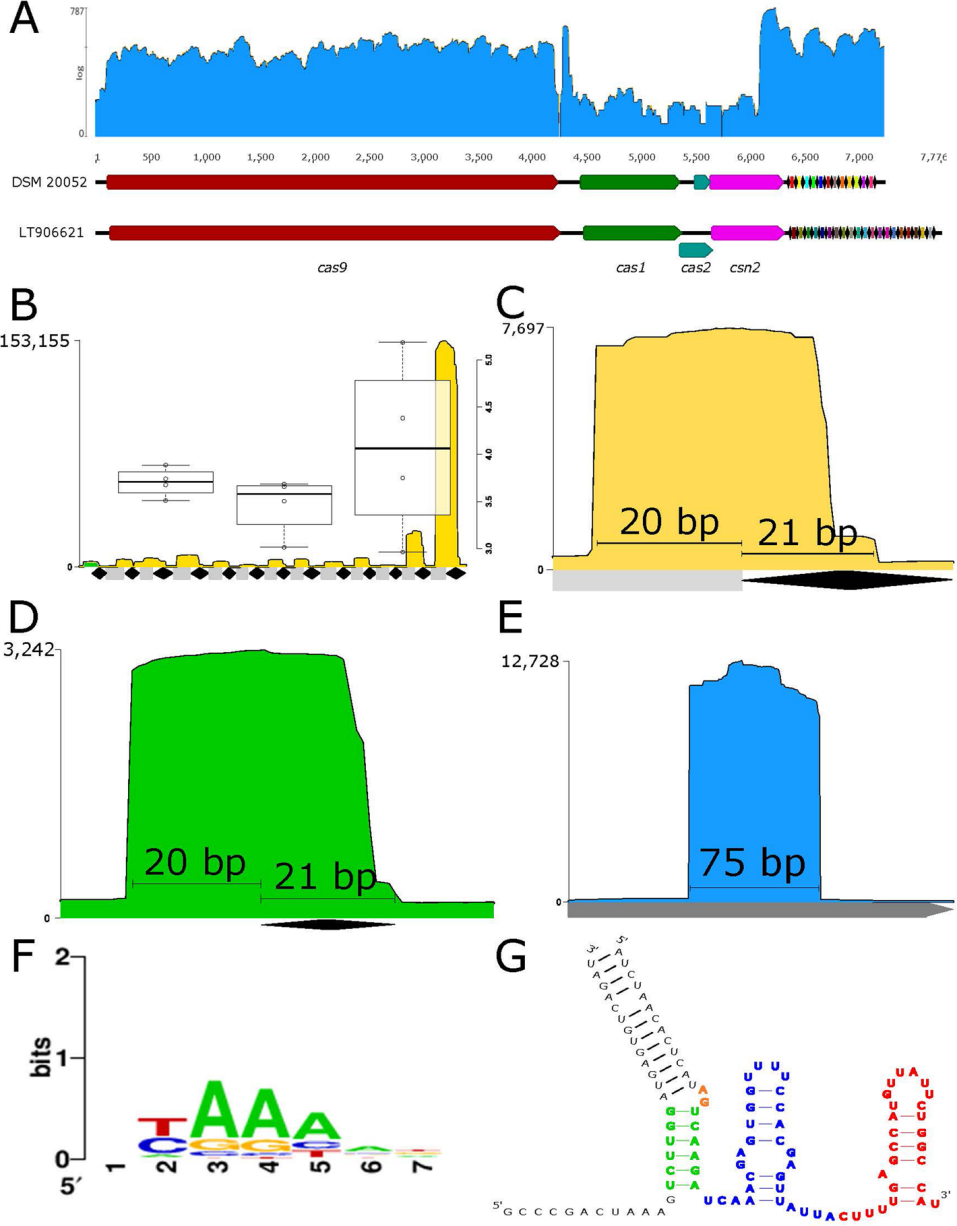
DSM 20052 CRISPR transcription. **a** Loci comparison of the putative Type II-A CRISPR-Cas loci in DSM 20052 (top) and LT906621 (bottom). *cas* genes are annotated. The repeat spacer arrays are visualized with the standard diamond/rectangle scheme. mRNA data for DSM 20052’s loci is overlaid. smRNA-seq expression profiles for the (**b**) repeat spacer array, (**c**) crRNA (yellow), (**d**), ldrRNA (green), and (**e**) tracrRNA (blue), the sequencing coverage is on the y-axis. In (**b**), the right y-axis shows the log transformed coverage for the boxplots. The leader is highlighted in green. In (**c**), the spacer (rectangle) and repeat (diamond) boundaries are labelled. In (**d**), the leader and repeat (diamond) boundaries are labelled. In (**e**), the predicted boundary is in gray and the actual is labelled. In (**f**) WebLogo of the proposed PAM sequence for the system. **g** Predicted structure of the tracrRNA with the lower stem (green), bulge (yellow), nexus (blue), and hairpin (red) highlighted

Next, we characterized the *L. fermentum* DSM 20052 Type II CRISPR-Cas system. Of the strains used in the BRIG analysis, only IMD0 130,101 (LT906621) also coded a Type II system (Fig. 1). A comparison of the two strains’ Type II loci is found in Fig. 6a. Each strain has the following *cas* genes: *cas9, cas1, cas2,* and *csn2*. Cas9 is the signature protein for Type II systems and *csn2* is the genetic marker for subtype II-A [19]. There were eight more spacers in LT906621 (twenty) than DSM 20052 (twelve). The repeat sequences for both systems were the same, only differing in their ancestral repeats, which often acquires SNPs. mRNA-Seq expression was overlaid on DSM 20052’s locus to show active transcription of the *cas* genes (Fig. 6a).

Small-RNA-Seq and in silico predictions were used to further characterize *L. fermentum* DSM 20052’s CRISPR-Cas system (Fig. 6). Expression levels for the CRISPR array, CRISPR RNA (crRNA), leaderRNA (ldrRNA), and tracrRNA were determined as shown in Fig. 6b, c, d, and e, respectively. In the CRISPR locus, the last two crRNAs (most ancestral) were found to be the most highly expressed spacers in the cell. Boundaries were determined for the crRNA, ldrRNA, and tracrRNA. The crRNA was found to consist of a 21 bp section of the CRISPR repeat and a 20 bp section of spacer, which is common in Type II-A CRISPR-Cas systems [20, 21]. The ldrRNA contains a 21 bp portion of repeat and a 20 bp leader. The tracrRNA was found to be 75 bp, which was much shorter than predicted (Fig. 6e). The structure of the tracrRNA was determined using NUPAK (Fig. 6g). The tracrRNA sequence modules are colored as previously described [22]. *L. fermentum* DSM 20052’s tracrRNA consists of all expected modules and contains only a single hairpin. Examining the BLAST results of *L. fermentum*’s Type II spacers, we predicted the PAM of DSM 20052 to be (C/T) AAA (Fig. 6f). Finally, a BLASTp comparison between *L. fermentum* DSM 20052’s Cas9 gene sequence, the *Streptococcus thermophilus* (Sth) Cas9 gene sequence, and the *Streptococcus pyogenes* (Spy) Cas9 gene sequence found at most only 32% AA identity between *L. fermentum* DSM 20052’s Cas9 and the other Cas9s. *L. fermentum* DSM 20052’s Cas9 is 1378 AAs long and its closest relatives are *Lactobacillus gorillae* and *Lactobacillus mucosae,* with 72 and 57% identity, respectively.

## Discussion

In this study, we genetically assessed the *Lactobacillus fermentum* species with focus on the type strain DSM 20052. Improving and polishing the previously published genome sequence of *L. fermentum* DSM 20052 allowed us to set a baseline genomic analysis for the type strain. The GC content (52.50%) is higher than what is typical for the low-GC *Lactobacillus* genus [23]. As lactobacilli are typically considered low-GC organisms, this finding may suggest that *L. fermentum* has seen less genomic drift. It is generally believed that as *Lactobacillus* species become more adapted to their environment, they begin to undergo genome decay [24]. Typically, lactobacilli with more than one niche have larger genomes and have undergone less genome decay. This is corroborated by a recent study looking at niche-adaptations in *Lactobacillus*; *L. fermentum,* while included in the study, did not have enough information to assign it a particular niche category [25]. This could imply that *L. fermentum* is a member of various niches and is still in the process of active adaptation. The portion (15%) of unknown/hypothetical genes certainly implies that there is still much to discover about *L. fermentum* DSM 20052. A few loci of interest were identified. A predicted *exopolysaccharide* gene has implications in food manufacturing for texture, in human health for biofilm formation, and in biotechnology for pathogen exclusion [26–28]. A putative CRISPR-Cas locus was also identified and will be discussed in depth below. As antibiotic resistance genes are raising concerns in both health and biotechnology applications, we examined *L. fermentum* DSM 20052 for any predicted antibiotic resistance genes and found none.

After examining the genome of *L. fermentum* DSM 20052, we performed a global phylogeny of *L. fermentum* using 38 genomes (Fig. 1)*.* This analysis revealed a great deal of diversity among *L. fermentum* strains. Nine clades were identified, with *L. fermentum* DSM 20052 as a part of a four-member clade, consisting of the strains HFB3 (LJFJ01.1), L930BB (NZ_CBUR), and Lfu21 (NZ_PNBB). Even though *L. fermentum* DSM 20052 was isolated from fermented beets, its clade members were isolated from human feces/colon biopsies. We would anticipate that related strains would have similar isolation sources. Since this is not the case for *L. fermentum* DSM 20052, this could imply *L. fermentum* enters the human microbiome through food sources and is a transient member (allochthonous), rather than a permanent member of the human microbiome (autochthonous). This fits with data found in IMNGS databases that show *L. fermentum*’s main environments to be food and human gut metagenomes. As transient members, it would also explain why *L. fermentum* does not have a specific niche-adaptation [25]. This finding also reflects the low survival of the type strain under GIT conditions (unpublished data).

Next, we performed whole genome comparisons using BRIG and Mauve with *L. fermentum* DSM 20052 and other complete genomes. For the BRIG Analysis, six genomes, NC_017465, NC_021235, LT906621, NZ_CP021790, NZ_AP017973, and NZ_CP019030 were chosen due to their closed genome status, and as selected representatives of distinct phylogenetic clades from Fig. 1. Their average genome size and GC% is 2.07 Mb and 51.6%, respectively, making *L. fermentum* DSM 20052 slightly smaller (1.89 Mb) and have a slightly higher GC% (52.5%) as compared to the other strains in the analyses. As seen in Fig. 2, comparing the seven strains via BRIG revealed three genomic islands in *L. fermentum* DSM 20052 that are absent in the other *L. fermentum* genomes. These islands are identifiable not only based on their absence in the other strains, but by a corresponding decrease in GC content. Further examination revealed that transposases and mobile genetic elements were frequently in and around these islands, which is indicative of acquired genes—potentially through horizontal gene transfer. No other loci of interest were identified (Additional files 1, 2, 3). Another *L. fermentum* DSM 20052 island encompassed the CRISPR locus - which is absent in the other genomes - with the exception of LT906621. A continuing examination of GC dips resulted in the identification of several other smaller GC dips in the BRIG alignment, which again correlated mostly with transposases and minor assembly gaps. These loci were often absent in the other *L. fermentum* genomes. We next analyzed gene synteny using whole genome MAUVE analysis (Fig. 3). Due to the large number of transposons identified in the BRIG analysis, we elected to include all completed genomes in the MAUVE analysis. Typically, the strains of a species are highly similar, and this manifests as large blocks of co-linearization in the MAUVE alignment. However, our analysis showed only small blocks of similarity and many rearrangements, indicating less conserved regions as compared to other *Lactobacillus* that are highly conserved and co-linear. This is unsurprising given the large number of MGEs discovered in *L. fermentum* DSM 20052. We were able to show that many of the small blocks identified by MAUVE remained in the same order and could be considered larger blocks of synteny (Fig. 3). Interestingly, genomes NZ_CP019030, NZ_CP021790, and NZ_CP017151 showed very little commonalities with the other *L. fermentum* genomes. While this could be a reflection of MGE’s, it may also imply inaccurate assemblies.

As CRISPR-Cas systems are a valuable reservoir of Cas-based genome editing technologies, we determined the occurrence and diversity of CRISPR systems in the thirty-eight analyzed *L. fermentum* genomes. On a species level, we found that 71.8% of strains encoded a predicted CRISPR system (Fig. 1). This is higher than *Lactobacillus* in general (62.9%), and bacteria as a whole (46%), suggesting that *L. fermentum* is a potential reservoir for novel CRISPR-based tools [6]. Type I is the most common system found in *L. fermentum* (53.8%), which reflects the overall dominance and diversity of Type I systems in nature [29]. Type I CRISPR-Cas systems have recently been studied for antimicrobial properties, and as such *L. fermentum* could be potentially explored as a programmed antimicrobial in microbiome settings [30]. While Type I is more common than Type II systems, it is the Type II’s signature Cas9 programmable endonuclease that is the most popular tool of the CRISPR toolbox [31]. 41% of *L. fermentum* strains contain a predicted Type II system. This is slightly higher than the Type II occurrence rate in lactobacilli (36%) and much higher than the occurrence rate in all bacteria (5%) [6, 32]. It is of note that one genome (OKQY01.1) was predicted to contain a Type I, II, and III system-- a rare occurrence [33]. Of the strains chosen for whole genome comparisons, all contained a putative Type I system except for *L. fermentum* DSM 20052, and only *L. fermentum* DSM 20052 and LT906621 contained a putative Type II system. The high level of CRISPR-Cas system occurrence and diversity is in line with the genomic diversity observed in the whole genome comparisons discussed in the previous section. A global analysis of the spacers found in *L. fermentum* revealed greater diversity than expected (Fig. 4). Typically, strains of a species have similar spacer history, or, “vaccination records,” resulting in the sharing of spacers, especially towards the ancestral ends of loci. In our analysis, we found only a limited number of shared spacers. Of the predicted Type II systems, NZ_CP021104’s, CP020353’s, NZ_CP011536’s, and NZ_PNBB’s loci shared common history, specifically in the ancestral spacers. However, there were several deletions or additional spacers in each locus, making the shared spacers a minority. With the exception of NZ_PNBB, these genomes were found in the same clade (Fig. 1). In contrast, the genomes with the most similar predicted records were NZ_AVAB, NC_010610, and NC_017465. All three putative Type I loci in each strain shared the same vaccination record as the other strains, with the exception of one or two spacers. Intriguingly, these genomes did not share clades (Fig. 1). Although there was not much congruity in the spacers, the predicted repeats of the *L. fermentum* CRISPR loci did share a high degree of similarity (Fig. 5). Taken together, these results illustrate how diverse *L. fermentum* species are, not only in terms of CRISPR systems but also in terms of genomic rearrangements. The high level of spacer diversity, especially with those strains isolated from similar origins, indicates varying evolutionary histories and exposures to different conditions. This could imply a wider range of habitats than originally thought for *L. fermentum* and provides a possible explanation for the high level of diversity as each strain would try to optimize to its niche.

We then performed an in-depth in silico analysis of *L. fermentum* DSM 20052’s putative CRISPR loci and revealed it to be a Type II-A, as evidenced by the *csn2* gene (Fig. 6a). As the only other strain with a putative Type II CRISPR-Cas system from those genomes selected for BRIG comparison, *L. fermentum* LT906621 was used to compare CRISPR loci. Both predicted systems were Type II-A, with *L. fermentum* LT906621 coding for a slightly larger CRISPR array. The repeats for each strain were identical, but they shared no common spacers. We also examined the expression levels of *L. fermentum* DSM 20052’s putative CRISPR loci using mRNA and smRNA-seq. mRNA expression levels showed that the *cas* genes are transcribed in *L. fermentum* DSM 20052. Analysis of expression in the CRISPR array using smRNA-seq revealed that the two most ancestral crRNA are the most highly expressed in *L. fermentum* DSM 20052’s CRISPR locus. This is highly unusual, as the newly acquired crRNA are typically the most highly expressed since they are more recently exposed to infection [34, 35]. It is possible that there is an internal promoter driving the expression of the ancestral crRNAs, and thus why the expression does not fit canonical expectations. The crRNA, ldrRNA, and tracrRNA had similar sizes as reported previously in lactobacilli (Fig. 6b-d) [20]. The in silico prediction of the tracrRNA was longer than the true boundaries predicted through smRNA-Seq, which has been previously reported [20]. This implies that our predictions are conservative compared to what is used in vivo*.* The tracrRNA structure showed the appropriate modules including the lower stem, bulge, upper stem, nexus, and contained a single hairpin (Fig. 6g). Finally, we predicted *L. fermentum* DSM 20052’s PAM to be (C/T) AAA (Fig. 6f). It is similar to several predicted PAMs in *L. gasseri* (TAA) [36]. Overall, expression for *L. fermentum* DSM 20052’s CRISPR loci fit canonical expectations, with the exception of the highly transcribed ancestral spacers. Despite its similarities to canonical Type II loci, the Cas9 in *L. fermentum* DSM 20052 is unique, only sharing 32% AA identity with either Sth’s or Spy’s Cas9—two of the most commonly used Cas9s in genome editing. This is especially intriguing as the Cas9s of Sth and Spy only share ~ 32% AA identity with each other. This marks *L. fermentum* DSM 20052 as a potential new orthogonal Cas9 for tool development.

## Conclusions

Overall, this study provides a basis for genetic analyses of *L. fermentum* strains, with an emphasis of the type strain DSM 20052. We determined the complete genome sequence of the type strain and carried out comparative genomic analyses revealing high variability within the species, encompassing MGEs and genomic islands. This genetic variability is also illustrated by the occurrence and diversity of hypervariable CRISPR-Cas systems. These observations highlight the value of determining the complete genome sequence of reference and type strains within a species, along with opening new avenues for the functional study of *Lactobacillus fermentum* strains and related species, and future exploration of valuable phenotypes.

## Materials and methods

### Genome sequencing

Long and short reads were generated for *L. fermentum* DSM 20052, the species type strain originally isolated from fermented beets, which was obtained from the American Type Culture Collection under strain reference ATCC 14931. PacBio sequencing was performed by RTL Genomics (Texas, US). DNA was extracted using Qiagen’s MagAttract HMW DNA Kit with the following modifications: sample was incubated at 37 °C, shaking (900 RPM) overnight with the addition of lysozyme and 6 μL of mutanolysin (20 μg/μL), then eluted with Tris-acetate-EDTA (TAE). Quality check was performed using dsDNA Broad Range DNA kit on the Qubit Fluorometer 3.0 and Fragment Analyzer by Advanced Analytical Technologies (Iowa, US) with the High Sensitivity Large Fragment 50 KB Analysis kit. Library preparation was performed from SMRTbell Libraries using PacBio Barcoded Adapters for Multiples SMRT sequencing with the following modifications: samples were pooled equimolar, 500 ng per sample of DNA were used, ligation was overnight, and final elution was 12 μL elution buffer. dsDNA High Sensitivity DNA kit on the Qubit Fluorometer 3.0 and Fragment Analyzer using High Sensitivity Large Fragment 50 KB Analysis Kit were used to perform library QC. Library preparation for sequencing was performed following PacBio’s protocol with a pre-extension time of 120 min and final loading of 6 pM. Short reads were generated by CoreBiome, Inc. (MN, USA). DNA was extracted using Qiagen’s MO Bio PowerFecal for high throughput on QiaCube with bead beating in 0.1 mm glass bead plates. Invitrogen’s Qiant-iT Picogreen dsDNA Assay was used to quantify DNA. Library preparation was completed using an adapted procedure from Illumina’s Nextera Library Prep Kit. Sequencing took place on an Illumina NextSeq using paired-end 2 × 150 reads and Illumina’s NextSeq 500/550 High Output V2 kit. Sequence Quality Control was set to filter a Q-Score < 20 and length < 50; cutadapt (v.1.15) was used to trim adapter sequences. SPAdes (v3.11.0) was used to assemble contigs and QUAST (v4.5) analysis was performed on contigs greater than 1000 bases. Short and long reads were then combined using Unicycler with default options. Remaining contigs were then hand-curated and joined using primer walking. The genome sequence was annotated using Rapid Annotations Subsystems Technology (RAST) [37]. The genome sequence was deposited at NCBI under Project PRJNA545488. Genomic features can be found in Table 1. Clusters of Orthologous Groups (COG) annotations were determined using eggnog-mapper, based on eggnog 4.5 data [38, 39]. ResFinder v 3.1 was used to search for antibiotic resistance genes [40].

### Comparative genomic analyses

Thirty-eight *L. fermentum* strains were selected for phylogenetic analyses (Table 1). A phylogeny was developed using the glycolysis gene phosphoglucomutase as its basis, following a previously proposed methodology [23]. Of the studied glycolysis genes, it was previously established that phosphoglucomutase would provide a highest degree of granularity in general and for high GC-content lactobacilli in particular [23]. After extracting the phosphoglucomutase gene sequence, nucleotide sequences were aligned using MUSCLE (maximum iteration was eight) [41]. Trees were then generated using RAxML (CAT GTR, Bootstrap using rapid hill climbing with random seed 1, and 100 replicates) [42]. A consensus tree was generated using a 50% threshold. Metadata was added to the cladogram using CLC Genomics (https://www.qiagenbioinformatics.com/).

Seven *L. fermentum* genomes were used for whole genome comparisons (DSM 20052, LT906621, NZ_AP017973, NZ_CP019030, NZ_CP021790, NC_021235, and NC_017465). A BRIG image was generated using BLAST Ring Image Generator (BRIG, 0.95), following parameters outlined in the manual [43]. A MAUVE alignment using all complete genomes was generated using default settings [44].

### Identification and annotation of CRISPR-Cas systems

Potential CRISPR loci were identified in 38 *L. fermentum* strains using the CRISPR recognition tool (CRT) [45]. Each predicted CRISPR-Cas system was then hand-curated for integrity, content, and assigned a type. Spacer visualization was achieved using CRISPRviz with standard options [46]. mRNA and smRNA were used to analyze transcriptional profiles of the CRISPR loci in DSM 20052. Cells were grown to mid-log phase and flash-frozen. Total RNA was extracted using Zymo Direct-Zol Miniprep kit (Zymo Research, Irvine, CA) according to a previously described protocol [47]. Library preparation and sequencing were performed by the Roy J. Carver Biotechnology Center from the University of Illinois (Urbana-Champaign, IL) using an Illumina HiSeq2500. Data was uploaded into Geneious (v. 11.1.5, https://www.geneious.com). Reads were then processed by trimming to an error probability limit of 0.001 and filtered to exclude reads less than 10 nt (smRNA) or a range of 28–150 nt (mRNA). Reads were mapped to the reference genome using Bowtie2 [48]. *trans*-activating-crRNA (tracrRNA) prediction was performed as previously described [49]. Briefly, we searched for the five modules of tracrRNA and the terminal GC-rich hairpins. Protospacer adjacent motif (PAM) prediction was carried out as previously described [20]. Briefly, protospacer hits were determined by BLASTing spacers against publicly available datasets. The flanking regions of positive hits were then used to identify sequence motifs.

## Supplementary information

**Additional file 1. **Results of a NCBI Conserved Domain Search of the GC island at 180kpb in *L. fermentum* DSM 20052.

**Additional file 2. **Results of a NCBI Conserved Domain Search of the GC island at 760kpb in *L. fermentum* DSM 20052.

**Additional file 3. **Results of a NCBI Conserved Domain Search of the GC island at 1550kpb in *L. fermentum* DSM 20052.

## Data Availability

The genomes generated and/or analyzed during the current study are available at NCBI under Project PRJNA545488 https://www.ncbi.nlm.nih.gov/bioproject/PRJNA545488/.

## Abbreviations

BLAST: Basic local alignment search tool
BRIG: BLAST ring image generator
COG: Cluster of orthologous groups
CRISPR: Clustered regularly interspaced short palindromic repeats
CRT: CRISPR recognition tool
GRAS: Generally recognized as safe
IMNGS: Integrated microbial next generation sequencing
LAB: Lactic acid bacteria
MGA: Mobile genetic element
QPS: Qualified presumption of safety
RAST: Rapid annotation using subsystem technology
SNP: Single-nucleotide polymorphism
