# Supplementary material for: Genomic characterization of Lactobacillus fermentum DSM 20052
Source: BMC Genomics. 2020 Apr 29;21:328. doi: 10.1186/s12864-020-6740-8 (PMC7191730; doi:10.1186/s12864-020-6740-8)
Supplement: Supplementary file 1 — Additional file 1. Results of a NCBI Conserved Domain Search of the GC island at 180kpb in L. fermentum DSM 20052. [file 12864_2020_6740_MOESM1_ESM.pdf]

List of domain hits

	Name	Accession	Description	Interval	E-value
[+]	HTH_21 super family	cl26233	HTH-like domain; This domain contains a predicted helix-turn-helix suggesting a DNA-binding ...	1132-1959	1.47e-131
[+]	Glyco_tranf_GTA_type super family	cl11394	Glycosyltransferase family A (GT-A) includes diverse families of glycosyl transferases with a ...	1-525	3.70e-60
[+]	BaeS	COG0642	Signal transduction histidine kinase [Signal transduction mechanisms];	13504-14502	1.10e-49
[+]	AzlC	pfam03591	AzlC protein;	16210-16629	3.76e-28
[+]	AzlD	pfam05437	Branched-chain amino acid transport protein (AzlD); This family consists of a number of ...	17086-17415	9.21e-12
[+]	Abhydrolase super family	cl21494	alpha/beta hydrolases; A functionally diverse superfamily containing proteases, lipases, ...	19477-19746	4.25e-08
[+]	Transposase_mut super family	cl27632	Transposase, Mutator family;	5855-6979	8.15e-113
[+]	OmpR	COG0745	DNA-binding response regulator, OmpR family, contains REC and winged-helix (wHTH) domain ...	12485-13168	3.00e-77
[+]	Tra8	COG2826	Transposase and inactivated derivatives, IS30 family [Mobilome: prophages, transposons];	2096-2602	3.77e-45
[+]	Stealth_CR2	pfam11380	Stealth protein CR2, conserved region 2; Stealth_CR2 is the second of several highly conserved ...	4889-5203	3.75e-38
[+]	Glyco_tranf_GTA_type	cd00761	Glycosyltransferase family A (GT-A) includes diverse families of glycosyl transferases with a ...	3908-4138	2.84e-08
[+]	WcaA	COG0463	Glycosyltransferase involved in cell wall bisynthesis [Cell wall/membrane/envelope biogenesis]; ...	3899-4768	8.57e-06
[+]	Stealth_CR1 super family	cl25328	Stealth protein CR1, conserved region 1; Stealth_C1 is the first of several highly conserved ...	4790-4861	8.28e-04
[+]	DacC	COG1686	D-alanyl-D-alanine carboxypeptidase [Cell wall/membrane/envelope biogenesis];	14673-15794	1.53e-57
[+]	MATE_like super family	cl09326	Multidrug and toxic compound extrusion family and similar proteins; The integral membrane ...	7131-8339	2.05e-24
[+]	HTH super family	cl21459	Helix-turn-helix domains; A large family of mostly alpha-helical protein domains with a ...	819-980	3.63e-04
[+]	Transposase_mut super family	cl27632	Transposase, Mutator family;	10623-10937	7.86e-29
[+]	Transposase_mut super family	cl27632	Transposase, Mutator family;	8964-9278	4.25e-15
[+]	DUF1828	pfam08861	Domain of unknown function DUF1828; This presumed domain is functionally uncharacterized.	11468-11662	4.24e-11
[+]	Cfa	COG2230	Cyclopropane fatty-acyl-phospholipid synthase and related methyltransferases [Lipid transport ...	20242-21069	7.09e-112
[+]	Transposase_mut super family	cl27632	Transposase, Mutator family;	9379-10491	1.11e-104
[+]	Cupin_5	pfam06172	Cupin superfamily (DUF985); Family of uncharacterized proteins found in bacteria and ...	11866-12270	3.81e-53
[+]	Transposase_mut super family	cl27632	Transposase, Mutator family;	10945-11364	2.18e-35

Additional File 1 | GC Island at 180kpb. Results of a NCBI Conserved Domain Search of the GC island at 180kpb in *L. fermentum* DSM 20052.
