# Supplementary material for: Genomic characterization of Lactobacillus fermentum DSM 20052
Source: BMC Genomics. 2020 Apr 29;21:328. doi: 10.1186/s12864-020-6740-8 (PMC7191730; doi:10.1186/s12864-020-6740-8)
Supplement: Supplementary file 2 — Additional file 2. Results of a NCBI Conserved Domain Search of the GC island at 760kpb in L. fermentum DSM 20052. [file 12864_2020_6740_MOESM2_ESM.pdf]

List of domain hits

Name	Accession	Description	Interval	E-value
infB	PRK05306	translation initiation factor IF-2; Validated	38071-40407	0e+00
PRK09194	PRK09194	prolyl-tRNA synthetase; Provisional	20635-22341	0e+00
truB	PRK01550	tRNA pseudouridine synthase B; Provisional	40855-41748	5.34e-169
PRK12838	PRK12838	carbamoyl phosphate synthase small subunit; Reviewed	31381-32433	2.61e-163
pyrH	PRK00358	uridylate kinase; Provisional	16336-17031	4.99e-130
SDH_alpha super family	cl12120	Serine dehydratase alpha chain; L-serine dehydratase (EC:4.2.1.13) is a found as a heterodimer ...	1969-2853	3.71e-87
TrmN6	COG4123	tRNA1(Val) A37 N6-methylase TrmN6 [Translation, ribosomal structure and biogenesis];	13300-14064	1.70e-78
RseP super family	cl28338	Membrane-associated protease RseP, regulator of RpoE activity [Posttranslational modification, ...	19360-20586	2.69e-70
PRK00092	PRK00092	ribosome maturation protein RimP; Reviewed	35698-36162	1.13e-59
CTP_transf_1	pfam01148	Cytidylyltransferase family; The members of this family are integral membrane protein ...	18523-19296	1.24e-53
rbfA	PRK00521	ribosome-binding factor A; Validated	40423-40782	3.00e-42
SDH_beta super family	cl27284	Serine dehydratase beta chain; L-serine dehydratase (EC:4.2.1.13) is a found as a heterodimer ...	1300-1767	1.62e-38
HTH_XRE	cd00093	Helix-turn-helix XRE-family like proteins. Prokaryotic DNA binding proteins belonging to the ...	8515-8685	1.09e-06
polC	PRK00448	DNA polymerase III PolC; Validated	22502-26830	0e+00
carB	PRK05294	carbamoyl phosphate synthase large subunit; Reviewed	32444-35266	0e+00
rpsB	PRK05299	30S ribosomal protein S2; Provisional	14489-15262	2.68e-157
nusA	PRK12327	transcription elongation factor NusA; Provisional	36209-37351	5.41e-151
HMG_CoA_synt_C super family	cl27874	Hydroxymethylglutaryl-coenzyme A synthase C terminal;	9638-10777	4.67e-147
tsf	PRK09377	elongation factor Ts; Provisional	15359-16228	1.42e-128
PRK05627	PRK05627	bifunctional riboflavin kinase/FMN adenylyltransferase; Reviewed	41822-42694	4.24e-103
Tra8	COG2826	Transposase and inactivated derivatives, IS30 family [Mobilome: prophages, transposons];	3548-4438	2.67e-71
TRI12 super family	cl27908	Fungal trichothecene efflux pump (TRI12); This family consists of several fungal specific ...	42863-44062	2.03e-65
GIY-YIG_UPF0213	cd10456	The GIY-YIG domain of uncharacterized protein family UPF0213 related to structure-specific ...	14057-14266	2.35e-26
DUF896	pfam05979	Bacterial protein of unknown function (DUF896); In B. subtilis, one small SOS response operon ...	11960-12145	4.75e-22
YlxR	cd00279	Ylxr homologs; group of conserved hypothetical bacterial proteins of unknown function; ...	37469-37702	1.28e-20
Phage_pRha	pfam09669	Phage regulatory protein Rha (Phage_pRha); Members of this protein family are found in ...	8795-8989	2.68e-14
PRK14830	PRK14830	undecaprenyl pyrophosphate synthase; Provisional	17745-18491	3.46e-138
frr	PRK00083	ribosome recycling factor; Reviewed	17052-17597	2.90e-86
Ribosomal_L7Ae super family	cl00600	Ribosomal protein L7Ae/L30e/S12e/Gadd45 family; This family includes: Ribosomal L7A from ...	37749-38039	3.78e-27
PaaD	COG2151	Metal-sulfur cluster biosynthetic enzyme [Posttranslational modification, protein turnover, ...	2859-3161	4.74e-27
UPF0154	pfam03672	Uncharacterized protein family (UPF0154); This family contains a set of short bacterial ...	12282-12458	3.32e-18
COG2932 super family	cl28411	Phage repressor protein C, contains Cro/C1-type HTH and peptisase s24 domains [Mobilome: ...	7742-8176	2.60e-32
PlsC	COG0204	1-acyl-sn-glycerol-3-phosphate acyltransferase [Lipid transport and metabolism];	12608-13210	1.10e-31
Tra8	COG2826	Transposase and inactivated derivatives, IS30 family [Mobilome: prophages, transposons];	44402-44722	6.25e-25
GlpC	COG0247	Fe-S oxidoreductase [Energy production and conversion];	8999-9376	4.03e-17
HTH_XRE	cd00093	Helix-turn-helix XRE-family like proteins. Prokaryotic DNA binding proteins belonging to the ...	8144-8314	1.75e-10
argD	PRK02936	acetylornithine aminotransferase; Provisional	26989-28113	0e+00
OAT	cd02152	Ornithine acetyltransferase (OAT) family; also referred to as ArgJ. OAT catalyzes the first ...	28888-30042	4.83e-175
PRK00942	PRK00942	acetylglutamate kinase; Provisional	28174-28872	1.43e-74
argC	PRK00436	N-acetyl-gamma-glutamyl-phosphate reductase; Validated	30138-31142	4.44e-130
PRK00215	PRK00215	LexA repressor; Validated	11166-11762	5.46e-95
Tra8	COG2826	Transposase and inactivated derivatives, IS30 family [Mobilome: prophages, transposons];	44718-45293	9.46e-35
Ion_trans_2	pfam07885	Ion channel; This family includes the two membrane helix type ion channels found in bacteria.	6033-6254	6.07e-12

Additional File 2 | GC Island at 760kpb. Results of a NCBI Conserved Domain Search of the GC island at 760kpb in *L. fermentum* DSM 20052.
