# Supplementary material for: Genomic characterization of Lactobacillus fermentum DSM 20052
Source: BMC Genomics. 2020 Apr 29;21:328. doi: 10.1186/s12864-020-6740-8 (PMC7191730; doi:10.1186/s12864-020-6740-8)
Supplement: Supplementary file 3 — Additional file 3. Results of a NCBI Conserved Domain Search of the GC island at 1550kpb in L. fermentum DSM 20052. [file 12864_2020_6740_MOESM3_ESM.pdf]

List of domain hits

Name	Accession	Description	Interval	E-value
[+] Tra8	COG2826	Transposase and inactivated derivatives, IS30 family [Mobilome: prophages, transposons];	22645-23595	2.86e-61
[+] HTH_21 super family	cl26233	HTH-like domain; This domain contains a predicted helix-turn-helix suggesting a DNA-binding ...	4270-5085	6.70e-59
[+] Transposase_mut super family	cl27632	Transposase, Mutator family;	5136-5609	1.07e-35
[+] HTH_21 super family	cl26233	HTH-like domain; This domain contains a predicted helix-turn-helix suggesting a DNA-binding ...	6984-7529	5.36e-20
[+] Tra8	COG2826	Transposase and inactivated derivatives, IS30 family [Mobilome: prophages, transposons];	3438-3854	3.16e-14
[+] ligA	PRK07956	NAD-dependent DNA ligase LigA; Validated	30772-32772	0e+00
[+] FabI super family	cl27754	Enoyl-[acyl-carrier-protein] reductase (NADH) [Lipid transport and metabolism];	38041-38808	2.46e-144
[+] ECF_ATPase_1	TIGR04520	energy-coupling factor transporter ATPase; Members of this family are ATP-binding cassette ...	43672-44472	1.44e-128
[+] truA	PRK00021	tRNA pseudouridine synthase A; Validated	41248-41988	3.75e-118
[+] EcfT	COG0619	Energy-coupling factor transporter transmembrane protein EcfT [Coenzyme transport and ...	42037-42795	2.41e-45
[+] HTH_21 super family	cl26233	HTH-like domain; This domain contains a predicted helix-turn-helix suggesting a DNA-binding ...	11116-11661	5.36e-20
[+] PRK15483	PRK15483	type III restriction-modification system StyLTI enzyme res; Provisional	16875-19841	0e+00
[+] gatA	PRK00012	aspartyl/glutamyl-tRNA amidotransferase subunit A; Reviewed	27732-29111	0e+00
[+] P-type_ATPase_HM	cd02079	P-type heavy metal-transporting ATPase; Heavy metal-transporting ATPases (Type IB ATPases) ...	9039-10826	0e+00
[+] PRK09219	PRK09219	xanthine phosphoribosyltransferase; Validated	36447-37013	1.13e-103
[+] rplM	PRK09216	50S ribosomal protein L13; Reviewed	40680-41108	7.35e-83
[+] FlgJ	COG1705	Flagellum-specific peptidoglycan hydrolase FlgJ [Cell wall/membrane/envelope biogenesis, Cell ...	37014-37619	9.15e-60
[+] Mod	COG2189	Adenine specific DNA methylase Mod [Replication, recombination and repair];	20490-21725	4.08e-41
[+] pcrA	TIGR01073	ATP-dependent DNA helicase PcrA; Designed to identify pcrA members of the uvrD/rep subfamily. ...	32822-35059	0e+00
[+] gatB	PRK05477	aspartyl/glutamyl-tRNA amidotransferase subunit B; Validated	26264-27676	0e+00
[+] PRK13055	PRK13055	putative lipid kinase; Reviewed	25235-26239	0e+00
[+] TrmA	COG2265	tRNA/tmRNA/rRNA uracil-C5-methylase, TrmA/RlmC/RlmD family [Translation, ribosomal structure ...	23771-25090	6.09e-157
[+] CamS	pfam07537	CamS sex pheromone cAM373 precursor; This family includes CamS, from which Staphylococcus ...	29678-30607	6.83e-130
[+] ECF_ATPase_2	TIGR04521	energy-coupling factor transporter ATPase; Members of this family are ATP-binding cassette ...	42839-43663	1.27e-118
[+] NirB super family	cl26176	NAD(P)H-nitrite reductase, large subunit [Energy production and conversion];	1859-3184	2.11e-84
[+] rpsI	PRK00132	30S ribosomal protein S9; Reviewed	40262-40651	1.55e-70
[+] PurK super family	cl27718	Phosphoribosylaminoimidazole carboxylase (NCAIR synthetase) [Nucleotide transport and ...	35483-36397	6.60e-43
[+] Crp	COG0664	cAMP-binding domain of CRP or a regulatory subunit of cAMP-dependent protein kinases [Signal ...	7706-8302	2.97e-33
[+] E1-E2_ATPase super family	cl27747	E1-E2 ATPase;	8711-8875	1.44e-04

Additional File 3 | GC Island at 1550kpb. Results of a NCBI Conserved Domain Search of the GC island at 1550kpb in *L. fermentum* DSM 20052.
